# The effect of patent ductus arteriosus treatment with paracetamol on pulmonary vascular resistance

**DOI:** 10.1038/s41372-022-01410-9

**Published:** 2022-05-18

**Authors:** Claire Murphy, Neidin Bussmann, David Staunton, Naomi McCallion, Orla Franklin, Afif EL-Khuffash

**Affiliations:** 1 Department of Neonatology, The Rotunda Hospital, Dublin, Ireland; 2 Department of Paediatrics, School of Medicine, Royal College of Surgeons in Ireland, Dublin, Ireland; 3 Department of Paediatric Cardiology, Children’s Health Ireland at Crumlin, Dublin, Ireland

**Keywords:** Congenital heart defects, Drug therapy

The use of paracetamol to achieve closure of a patent ductus arteriosus (PDA) in premature infants lacks robust safety data [1]. Paracetamol exerts its vasoconstrictive effect on ductal tissue by inhibition of prostaglandin H2 synthase, which is responsible for the metabolism of arachidonic acid [2]. Dysregulation of this pathway is implicated in the development of pulmonary arterial hypertension [3]. In this report, we assess the impact of paracetamol administration for PDA closure on pulmonary vascular resistance (PVR) in preterm infants <29 weeks gestation using echocardiography.

This is a report of fifteen infants <29 weeks of gestation who were enroled into the PDA RCT, a randomised controlled trial of PDA treatment, who received open label paracetamol for ductal closure beyond the first week with a dose of 15 mg/kg, four times a day for 5 days (ISRCTN:13281214) [4]. Ethical approval from the Rotunda Research Ethics Committee and written parental consent was obtained. Echocardiography studies were acquired prior to paracetamol and following completion of the treatment course. We obtained measurements of PDA characteristics, left (LV) and right ventricular (RV) function, and surrogate markers of PVR using the ratio of pulmonary artery acceleration time to right ventricular ejection time (PAATi) and LV eccentricity index (LV EI). Paired data were compared using the paired student *t* test or the non-parametric equivalent as appropriate. Categorical variables were compared using the Chi square or the Fisher’s exact test as appropriate.

Fifteen infants were included. Their median gestation, birthweight were 25.7 [25.3–26.6] weeks, 810 [705–995] g respectively. Eleven were male and ten were delivered by caesarean section. Paracetamol was commenced at a median of 15 [8–19] days. There was no change in the PDA diameter, LA:Ao or LVEDD. Descending aorta end diastolic flow changed from a retrograde to an ante grade direction over the two time points (Table 1). There was a decrease in PAATi and an increase in LV EI over the two time points (Table 1). One infant died from severe necrotising enterocolitis which occurred in the week following treatment. Five infants were in the intervention arm and received ibuprofen. Seven infants (47%) underwent a PDA ligation. Chronic lung disease occurred in 12 (80%) survivors.

**Table 1:** Pre- and post-paracetamol treatment echocardiography measurements.

	Pre-treatment	Post-treatment	*p*
Heart rate	163 ± 17	167 ± 14	0.52
PDA characteristics			
PDA diameter (mm)	3.2 ± 0.8	3.0 ± 0.7	0.31
PDA systolic:diastolic velocity ratio	5.6 [3.0–6.1]	3.6 [2.3–5.3]	0.06
LA:Ao	2.1 ± 0.4	1.9 ± 0.4	0.14
LV end diastolic diameter (mm)	15 ± 3	15 ± 3	0.97
Descending aorta EDV (m/s)	−0.12 [−0.21 to −0.07]	0.13 [0.06–0.19]	<0.01
Myocardial function			
LV ejection fraction (%)	60 ± 9	59 ± 8	0.97
RV fractional area change (%)	0.30 ± 0.11	0.29 ± 0.12	0.76
Surrogate PVR markers			
PAAT	44 ± 13	38 ± 9	0.10
PAATi	0.34 ± 0.09	0.25 ± 0.04	<0.01
LV Eccentricity Index	1.3 ± 0.1	1.4 ± 0.2	0.049

Values are presented as means ±(SD), medians [IQR] or count (%).

*PDA* Patent Ductus Arteriosus, *LA:Ao* Left atrial to aortic root ratio, *LV* left ventricle, *EDV* end diastolic velocity, *RV* right ventricle, *PAATi* Pulmonary Artery Acceleration Time indexed to heart rate.

None of the infants achieved ductal closure after treatment with paracetamol and there was no reduction in ductal calibre observed. Despite this, there was evidence of shunt volume modulation with an improvement in diastolic flow in the descending aorta. The underlying pathophysiological process to explain these findings likely involves the arachidonic acid pathway: inhibiting prostaglandin production results in an increase in vasomotor tone in the resistance-level pulmonary arteries. Changes within the AA pathway can result in production of COX- dependent vasoconstrictors (thromboxane) instead of COX-dependent vasodilators [3, 5]. The increase in pulmonary vasomotor tone could possibly explain the effect of paracetamol on ductal closure, by reducing the PDA shunt gradient, instead of reducing the diameter of the PDA. However, it is worth highlighting that other confounding factors, such a CLD evolution, may have contributed to the PVR changes in the cohort. Caution is required regarding the longer term effects of paracetamol on PVR.

## Data Availability

Data is unavailable for access as the data is confidential.
